# Identification and expression analysis of methyl jasmonate responsive ESTs in paclitaxel producing *Taxus cuspidata* suspension culture cells

**DOI:** 10.1186/1471-2164-13-148

**Published:** 2012-04-24

**Authors:** Sangram K Lenka, Nadia Boutaoui, Bibin Paulose, Kham Vongpaseuth, Jennifer Normanly, Susan C Roberts, Elsbeth L Walker

**Affiliations:** 1 Department of Biology, University of Massachusetts, Amherst, MA, 01003, USA; 2 Department of Biochemistry and Molecular Biology, University of Massachusetts, Amherst, MA, 01003, USA; 3 Department of Chemical Engineering, University of Massachusetts, Amherst, MA, 01003, USA; 4 Plant Biology Graduate Program, University of Massachusetts, Amherst, MA, 01003, USA

## Abstract

**Background:**

Taxol® (paclitaxel) promotes microtubule assembly and stabilization and therefore is a potent chemotherapeutic agent against wide range of cancers. Methyl jasmonate (MJ) elicited *Taxus* cell cultures provide a sustainable option to meet the growing market demand for paclitaxel. Despite its increasing pharmaceutical importance, the molecular genetics of paclitaxel biosynthesis is not fully elucidated. This study focuses on identification of MJ responsive transcripts in cultured *Taxus* cells using PCR-based suppression subtractive hybridization (SSH) to identify genes involved in global pathway control.

**Results:**

Six separate SSH cDNA libraries of paclitaxel-accumulating *Taxus cuspidata* P991 cell lines were constructed at three different post-elicitation time points (6h, 18h and 5 day) to identify genes that are either induced or suppressed in response to MJ. Sequencing of 576 differentially screened clones from the SSH libraries resulted in 331 unigenes. Functional annotation and Gene Ontology (GO) analysis of up-regulated EST libraries showed enrichment of several known paclitaxel biosynthetic genes and novel transcripts that may be involved in MJ-signaling, taxane transport, or taxane degradation. Macroarray analysis of these identified genes unravelled global regulatory expression of these transcripts. Semi-quantitative RT-PCR analysis of a set of 12 candidate genes further confirmed the MJ-induced gene expression in a high paclitaxel accumulating *Taxus cuspidata* P93AF cell line.

**Conclusions:**

This study elucidates the global temporal expression kinetics of MJ responsive genes in *Taxus* suspension cell culture. Functional characterization of the novel genes identified in this study will further enhance the understanding of paclitaxel biosynthesis, taxane transport and degradation.

## Background

Although the supply of most plant-derived secondary metabolic products is through harvest of the whole plant, many of these commercially valuable compounds are produced in slow-growing or non-agriculturally produced species [1]. Harvest from natural sources is often insufficient to provide adequate quantities of the product of interest in a sustainable manner. Plant cell culture provides an alternative production source in which environmental conditions can be more easily controlled, manipulated, and optimized to yield high quantities of these valuable products (*i.e.*, secondary metabolites).

The anticancer drug paclitaxel (registered as a trademark Taxol® by Bristol-Myers Squibb) is a secondary metabolite with a complex chemical structure that has been approved by the Food and Drug Administration (FDA) for the treatment of breast, ovarian and lung cancers, AIDS-related Kaposi's sarcoma as well as coronary artery disease ( http://www.fda.gov). The production of paclitaxel from *Taxus* cell cultures has been achieved by us and others [2-11]. Plant cell culture technology has received significant attention in meeting the demand for paclitaxel and this method of production of paclitaxel has been licensed by Bristol-Myers Squibb to Phyton Biotech Inc. (Fort Worth, TX).

Paclitaxel is a member of the taxane family of compounds that are produced by all species of yew. Over 400 taxanes have been reported from various *Taxus* species [12]. The structure of paclitaxel was determined by Wani *et al.*, (1971) as a diterpenoid (molecular formula of C_47_H_51_NO_14_) with a highly functionalized taxane skeleton [13]. Taxanes vary in the functional groups and side-chain components attached to the tricyclic taxane skeleton. Many steps of the biosynthetic pathway have been elucidated through the isolation of genes and enzymes from *Taxus* tissues and cultured cells [14-16]. Using molecular approaches, the genes encoding GGPP synthase [17], 10-deacetylbaccatin III-10-O-acetyl transferase [18], taxadiene synthase [19], cytochrome P450 taxane 10β-hydroxylase [20], 3'-N-debenzoyl-2'-deoxyTaxol N-benzoyltransferase [21], baccatin III:3-amino-3-phenylpropanoyl transferase [22], taxane 13α-hydroxylase [23], taxane 2α-O-benzoyltransferase [24], and taxa-4(20),11 (12)-dien-5α-ol-O-acetyl transferase [25] were identified.

The biotic elicitor methyl jasmonate (MJ) is an integral component of the signal transduction process that regulates the inducible defence systems of plants. Exogenous MJ is highly effective at eliciting secondary metabolite accumulation in a variety of plant cell culture systems [26]. Accordingly, paclitaxel accumulation is significantly enhanced upon MJ addition [27-31].

The non-model gymnosperm *Taxus* is evolutionally diverged and quite distantly related to model angiosperm species. This presents significant challenges to genomics approaches for understanding paclitaxel synthesis and regulation, and necessitates the generation of *Taxus*-specific genetic information in order to establish gene-to-metabolite links. Genetic information for *Taxus* species has begun to emerge from transcriptome sequencing of *Taxus* organs (needles, bark, cambial meristematic cells *etc*.) [32-34]. However, a differentially-regulated transcripome of paclitaxel accumulating cultured cells in controlled environmental conditions, and in response to secondary metabolite elicitors like MJ, needs to be characterized. Here we describe the results from transcript profiling experiments in *Taxus* culture cells to identify genes involved in global pathway control, including not only potential paclitaxel biosynthetic genes, but also genes that may be involved in MJ-signaling, taxane transport, or taxane degradation. Using a PCR-based suppression subtractive hybridization strategy, MJ responsive ESTs were isolated from the paclitaxel-accumulating P991 *Taxus cuspidata* cell line. Expression analysis of randomly selected differentially-regulated ESTs from the MJ-treated SSH libraries was also performed using macroarrays. Computational functional annotation and Gene Ontology (GO) analysis of up-regulated EST libraries show enrichment of several known paclitaxel biosynthetic genes as well as novel transcripts. RT-PCR analysis of a set of 12 candidate genes further confirmed the MJ-induced gene expression in the paclitaxel accumulating cell line. Hence, this study is useful in expanding our understanding of the molecular genetics of the complex paclitaxel biosynthesis process.

## Results

### Differential screening of MJ-responsive ESTs

P991 is a well characterized *T. cuspidata* cell line that produces higher amounts of taxanes after MJ elicitation [27,35]. This cell line was used to detect differentially expressed MJ responsive transcripts with a PCR-based cDNA subtraction method. Six distinct driver/tester combinations were used to construct six different SSH libraries. To generate libraries enriched for up-regulated genes, the combinations of driver/tester were as follows:

1) Driver = unelicited control at 6 hour time point; tester = MJ-elicited at 6 hour time point

2) Driver = unelicited control at 18 hour time point; tester = MJ-elicited at 18 hour time point

3) Driver = unelicited control at 5 day time point; tester = MJ-elicited at 5 day time point

In contrast, to produce SSH libraries enriched for down-regulated genes the opposite driver/tester combination (*i.e.* MJ-elicited driver and unelicited tester) was used with the same cDNA samples. Single pass sequencing was carried out on 288 recombinant clones each from the MJ up- and down-regulated SSH libraries (96 clones each from 6h, 18h and 5 day time point of both the up/down regulated libraries). Among the 576 clones sequenced, 548 were considered for contig assembly and annotation after excluding vector sequence contamination. All the EST sequences from the three up-regulated libraries were used together for contig assembly with CAP3. Similarly, the ESTs from the three down-regulated libraries were used together for asssembly of down-regulated contigs. CAP3 assembly of the ESTs generated 32 MJ up-regulated contigs, 123 singletons and 23 MJ down-regulated contigs, 153 singletons (Table 1) [36]. Hence, the up-regulated libraries contain 155 unigenes (combination of contigs, Table 2, Additional file 1: Table S1 and singletons, Supplemental 2) and 176 unigenes are represented in the down-regulated libraries ( Additional file 2: Table S2). The mean length of the ESTs obtained from all the libraries was 658 bp, which is adequate for sequence similarity based functional classification.

**Table 1:** **Overview *T. cuspidata *P991 SSH libraries**

**Features**	**Up-regulated libraries**	**Down Regulated libraries**
Number of clones sequenced	288	288
Number of clean ESTs	283	265
Number of non-redundant ESTs	155	176
Total number of contigs	32	23
Total number of singletons	123	153
Number of randomly chosen ESTs used for macroarray analysis	128	128

**Table 2:** **Identity and description of contigs obtained from *T. cuspidata *up-regulated SSH libraries**

**Contig**	**Accession No**	**Match with Accession No**	**Description**	**Query coverage (%)**	**E Value**
03	HE799317	ABR16435	Predicted Cytochrome P450	66	5.00E-72
08	HE799322	BAF46017	Putative class I chitinase	35	5.00E-64
21	HE799335	AAR13860	Taxadiene synthase	92	4.00E-61
25	HE799339	ABK26019	Unknown	34	1.00E-42
30	HE799344	BAD90813	Thaumatin-like protein	76	2.00E-37
04	HE799318	AAZ41362	Taxadiene synthase	94	5.00E-30
28	HE799342	XP_002264098	hypothetical protein	49	2.00E-27
26	HE799340	CAI56321	putative leucoanthocyanidin reductase	68	3.00E-25
18	HE799332	XP_002516771	putative Lipoxygenase	59	5.00E-25
15	HE799329	AAS89065	putative taxoid hydroxylase	65	8.00E-22
17	HE799331	ABK21418	Unknown	72	2.00E-23
29	HE799343	ACH59388	putative Chalcone synthase	36	3.00E-21
12	HE799326	AAO85809	putative S-adenosylmethionine synthetase	43	3.00E-20
09	HE799323	BAD10865	predicted 1-aminocyclopropane-1-carboxylic acid oxidase	79	7.00E-18
11	HE799325	ZP_03319418	Hypothetical protein	55	5.00E-16
01	HE799315	AAT73199	predicted taxoid acyltransferase	81	9.00E-16
14	HE799328	XP_002264858	Hypothetical protein	66	9.00E-16
07	HE799321	XP_002973748	Hypothetical protein (XP_002973748)	32	1.00E-15
16	HE799330	XP_001768181	similar to predicted protein (XP_001768181)	47	2.00E-15
27	HE799341	ABR16535	Unknown	16	7.00E-15
02	HE799316	ABR17205	putative DNA binding protein	79	2.00E-13
05	HE799319	ACN40018	Unknown	60	2.00E-09
32	HE799346	ADQ12769	Cytochrome P450	41	3.00E-09
06	HE799320	ABK22210	Unknown	39	8.00E-07
23	HE799337	ADK74829	putative Immunomodulatory protein	13	3.00E-05
20	HE799334	ABK23181	Unknown	11	3.00E-04
10	HE799324	ABK24846	putative Gibberellin 3-beta-hydroxylase	38	0.001
31	HE799345	ADM79415	similar to Dormancy/auxin associated-like protein	27	0.001
22	HE799336	YP_260089	Cytotoxin Mcf	41	0.003
19	HE799333	XP_002283086	hypothetical protein	8	0.005
13	HE799327	-	No hits in the database	-	-
24	HE799338	-	No hits in the database	-	-

### Functional classification of the unigenes

Putative functional annotation of the unigenes ( Additional file 2: Table S2) was assigned by BLASTX analysis against the GenBank non-redundant database for sequence similarity (E-value ≤ 10^−2^). Unigenes showing BLASTX homology at E >10^-2^ were designated as unigenes with no match in the database. Approximately 47% of the unigenes fell into this category, and may be specific to *Taxus* species. Approximately 12% of the sequences represented evolutionarily conserved proteins whose function is not known. Many of the known paclitaxel biosynthetic genes were induced, as expected. Several novel transcripts that are similar, but not identical, to known taxane biosynthetic enzymes were identified. These included two genes that are similar to known taxane acyl transferases and five genes that are related to known taxane hydroxylase. Experimental characterization of these novel genes would be needed to definitively determine the exact acylation or hydroxylation reactions catalyzed by the encoded enzymes.

Functional classification of MJ up-regulated unigenes was determined based on GO analysis for biological process, molecular function and cellular component ( Additional file 3: Figure S1a-c). Among the biological process category, unigenes belonging to oxidation-reduction processes (GO:0055114), responses to stimulus (GO:0050896), small molecule biosynthetic processes (GO:0044283) and paclitaxel biosynthetic processes (GO:0042617) were over-represented. In the molecular function category, oxidoreductase activity (GO:0016491) was most significantly enriched, followed by iron ion binding (GO:0005506) and transferase activity (GO:0016740). Finally, in the cellular component category, proteins associated with intracellular organelles (GO:0043229), membranes (GO:0016020) and cytoplasmic parts (GO:0044444) were most enriched. KEGG pathway analysis of the encoded proteins further indicated MJ-induced paclitaxel, L-methionine and oxylipin biosynthesis pathways in the up-regulated SSH libraries. There were too few truly down-regulated unigenes available (see below) for GO analysis of down regulated transcripts.

### Evaluation of MJ-responsive differential expression using macroarrays

**Figure 1:**
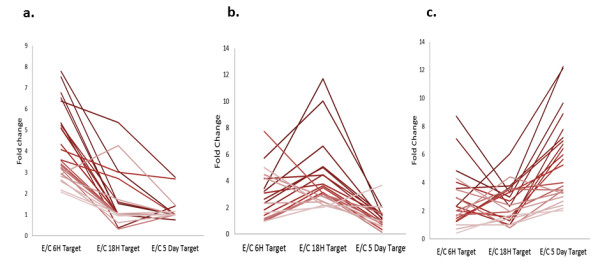
**Expression pattern of clones from up-regulated libraries.** Macroarrays spotted with randomly selected clones (probes) from all six SSH libraries were hybridized with labelled RNA targets prepared from mock elicited and elicited cells at three time points: 6h, 18h, and 5 day. Expression profile plots for the set of probes corresponding to each up-regulated library are shown: **a**, 6h library probes **b**, 18h library probes **c**, 5 day library probes. Expression is given as the fold-change in gene expression in the elicited culture over the un-elicited culture. Only results from probes showing a statistically significant change in expression (P < 0.05) are shown.

**Figure 2:**
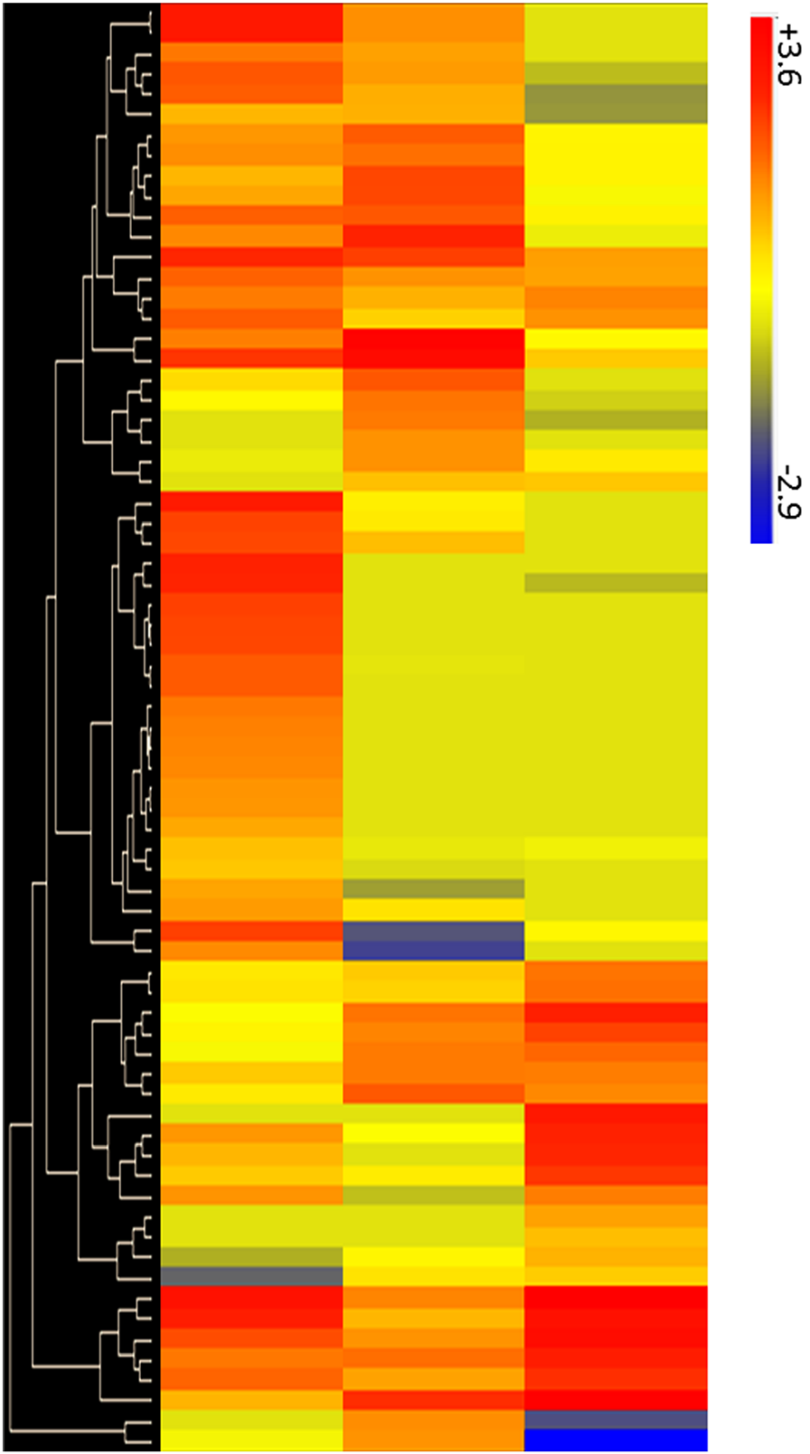
**Heat map showing overall expression patterns of differentially regulated genes from up-regulated libraries.** Hierarchical clustering of significantly MJ up-regulated genes was performed using average linkage and Euclidean distance as a measurement of similarity. Only results from probes showing a statistically significant change in expression for at least one time point (P < 0.05) are shown.

Probe sets designed from up- and down-regulated SSH libraries were spotted manually as macroarrays on nylon membranes and hybridized with different RNA targets. Of the 256 randomly chosen ESTs in the macroarray, 81 (71 up- and 10 down- regulated) ESTs showed a significant change in intensity following MJ elicitation ( Additional file 4: Table S3). One set of genes showed the highest up-regulation trend at early time points (6h and 18h) following MJ elicitation (Figures 1a, b and 2), while MJ responsive expression of a separate set of genes exhibited a difference in expression pattern at the late time point (5 day; Figures 1c, 2). On the other hand, the majority (92.2%) of MJ down-regulated unigenes were found to be false positive SSH clones ( Additional file 4: Table S3, Additional file 5: Figure S2, Additional file 6: Figure S3, Additional file 7: Figure S4) based on this analysis. Hierarchical clustering analysis of the temporal differential gene expression patterns of the individual ESTs in the macroarray (Figure 2) clearly demonstrated the distinctions between the MJ-responsive early, middle and late gene regulation.

### Confirmation of MJ-induced expression by RT-PCR

**Figure 3:**
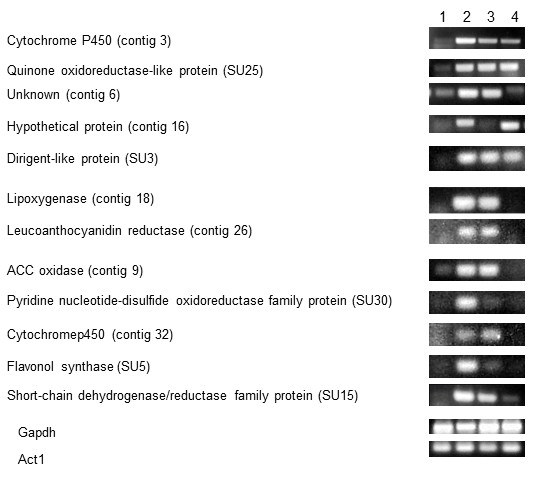
**Semi-quantitative RT-PCR analysis of 12 selected up-regulated unigenes.** Semi-quantitative RT-PCR was carried out using RNA from the *Taxus cuspidata* P93AF cell line. RNA samples were taken at 6h, 18h and 5 day after MJ elicitation, and from mock elicited cells at the same time points. Lane 1: Mock elicited for 18h, Lane 2-4: elicited with 100 μM MJ for 6h, 18h or 5 day, respectively. *Gapdh* and *Actin1* were used as normalization controls.

Twelve randomly selected genes from the up-regulated SSH library were evaluated for transcriptional response in another paclitaxel-accumulating line *T. cuspidata* P93AF using semi-quantitative RT-PCR. All 12 genes were up-regulated at 6h after MJ elicitation, further supporting the early overall response observed in the macroarray (Figure 3). Transcript levels for flavonol synthase (D5_88H) and the pyridine nucleotide-disulfide oxidoreductase family protein (18R_32H) went down after 6h. Cytochrome P450 (contig 32), unknown (contig 6), ACC oxidase (contig 9), leucoanthocyanidin reductase (contig 26) and lipoxygenase (contig 18) were elevated at both 6h and 18h. However, semi-quantitative RT-PCR could not detect transcripts of these genes on day 5 after elicitation. Transcripts encoding cytochrome P450 (contig3), quinone oxidoreductase-like protein (18R_44D), hypothetical protein (contig 16), dirigent-like protein (18R_17A) and short-chain dehydrogenase/reductase family protein were at elevated levels on day 5 as well as at early time points. Elevated expression of these randomly chosen sequences in the *T. cuspidata* P93AF cell line upon MJ-elicitation further supported that regulated genes were successfully identified using the subtracted cDNA libraries from cell line P991.

## Discussion

MJ potentiates secondary metabolite accumulation in a variety of plant suspension cell culture systems, and is now a widely-used augmenting tool for bio-active molecule production [26]. Accordingly, paclitaxel accumulation is significantly enhanced with MJ elicitation in *T. cuspidata* suspension cultured cells [27,37-40]. MJ is also implicated in specific induction of a wide range of secondary metabolite genes involved in biotic-stress response and wounding [41]. Conversely, primary metabolic genes such as those involved in photosynthesis, electron transport and cytoskeletal organization are either down-regulated or unaffected by MJ elicitation [42]. MJ elicitation and targeted cloning approaches have been effectively used in *Taxus* to identify specific taxane biosynthetic genes [43,44]. Therefore, an up-regulated cDNA library of MJ treated cells will presumably contain genes related to secondary metabolism, including paclitaxel biosynthesis.

SSH is a powerful genomics technique that enriches differentially regulated genes and has mostly been used to identify transcripts expressed in contrasting environmental conditions. SSH is a suitable approach in *Taxus* where the genomic information is currently scarce. We used the PCR-based SSH approach to identify MJ responsive (up- and down-regulated) transcripts that precede paclitaxel accumulation in the paclitaxel accumulating *T. cuspidata* P991 cell line. Putative up-regulation of as many as 155 unigenes and down-regulation of 176 unigenes of diverse functional groups suggested involvement of a spectrum of genes regulating paclitaxel biosynthesis, MJ-signaling and response, and potentially, taxane transport and taxane degradation. The up-regulated SSH library contained several previously known positively regulated genes that are likely to be directly involved in paclitaxel synthesis ( Additional file 2: Table S2). For instance, putative taxadiene synthase, taxane hydroxylase and taxane acyl transferases having strong sequence similarity with the corresponding known paclitaxel biosynthetic pathway genes were detected in the up-regulated library. Taxadiene synthase catalyses the first committed step of paclitaxel biosynthesis in *Taxus* by cyclization of the linear isoprenoid substrate geranyl geranyl diphosphate (GGPP) to form taxa-4(5),11(12)diene [19]. Four unigenes representing different isoforms of putative taxadiene synthase were up-regulated in the library. It will be interesting to unravel the individual contribution of each of these taxadiene synthase genes towards paclitaxel production. Another paclitaxel biosynthetic gene, taxoid 2-α-hydroxylase, produces 2α-hydroxy taxoids as starting materials for subsequent acylation at the C_2_-position of the taxane core that ultimately bears a benzoyl group, an important paclitaxel pharmacophore [45]. Five novel putative taxoid hydroxylases that are similar but not identical to known taxoid hydrolases (taxoid 2-alpha hydroxylase, taxoid 5-alpha hydroxylase, and taxoid 7-beta hydroxylase) were detected in the up-regulated library. Each might function to hydroxylate taxoids at specific positions. Similarly, novel taxane acyl transferases are not only important targets for genetic manipulation to improve paclitaxel production in genetically engineered host systems but also provides a means of attaching modified aroyl groups to taxoid precursors to improve drug efficacy [18,21,25,46]. Characterization of the two newly identified candidate acyl transferases in the library will further suggest roles for these novel enzymes in paclitaxel biosynthesis.

Apart from the paclitaxel biosynthesis genes, up-regulated genes that are potential candidates for regulation of the MJ response were also obtained. One such gene encodes ACC oxidase (1-aminocyclopropane-1-carboxylate oxidase),which is the final step in the synthesis of the plant hormone ethylene [47]. Ethylene is known to be produced following MJ elicitation, and to function in suppression of the MJ signal. An increased production of paclitaxel upon application of ethylene synthesis inhibitors to elicited *Taxus* cultures was reported by Zhang et al., (2003) [48]. Disruption of this ACC oxidase gene may allow the MJ signal to be strengthened, which may lead to increased paclitaxel accumulation.

The library was also enriched with up-regulated genes that presumably have no direct role in paclitaxel accumulation. Good examples are the putative class I chitinase, thaumatin (also known as PR-5); chalcone synthase, and defensin, all of which are defense-related genes that have well-characterized roles in model plant species [49,50]. These genes are known to be induced by MJ in other plant species and thus are unlikely to be related to the synthesis of taxanes, which are unique to *Taxus* species. For several transcripts, a reliable prediction of general biochemical activity can be made, but predictions neither suggest nor challenge a role in paclitaxel synthesis. Genes in this class include three cytochrome P450 genes that lack significant similarity to the several known taxane biosynthetic pathway P450s, two distinct lipoxygenase genes, and a few other enzyme encoding genes. Any of these genes could participate in the uncharacterized steps of the paclitaxel biosynthetic pathway (e.g., hydroxylation reactions and oxetane ring formation), but could equally well be unrelated to paclitaxel synthesis. Further characterization will be necessary to determine whether these genes have direct relevance to paclitaxel biosynthesis.

A large class of genes (59%) have functions that cannot be predicted based on sequence similarity information. These genes may be involved in important but uncharacterized processes of MJ-signaling, taxane transport or taxane degradation. As many as 47% of these ESTs showing no sequence homology with any of the available Genbank sequences may be specific to *Taxus* species. The other 12% of sequences are similar to genes described as encoding hypothetical proteins or proteins with unknown function. Enrichment of unigenes belonging to various biological process categories (GO), such as oxidation-reduction process (GO:0055114), response to stimulus (GO:0050896), small molecule biosynthetic process (GO:0044283) and paclitaxel biosynthetic process (GO:0042617), provide strong molecular evidence that the SSH-based MJ elicited transcript screening is a good method to identify genes related to paclitaxel metabolism. Nevertheless, this strategy did not enable the isolation of all the known paclitaxel metabolism genes indicating that the collection of MJ-inducible genes identified here is not comprehensive. Sequencing of the total MJ-induced transcriptome will be required to identify transcripts for every gene related to paclitaxel accumulation.

In addition to confirming up-and down-regulated genes, macroarrays identified false positive clones that appeared in the libraries but were not actually differentially regulated in response to MJ. Although most genes identified as being up-regulated were confirmed as such by macroarray analysis, the false positive rate in the down-regulated libraries was 92.2%. SSH libraries of highly complex eukaryotes have often been reported to contain high percentages (30-50%) of false positive clones that escaped subtraction [51,52]. These high false positive rates are usually attributed to non-specific annealing of primers, partial hybridization of driver that allows tester amplification and non-specific annealing of suppression adaptors [53]. While any of these could have been factors in producing the high false positive rate in our down-regulated libraries, we also note the possibility that there could simply be very few genes that are down-regulated by MJ in the *Taxus* cultures. If few or no down-regulated transcripts occur, then the SSH approach is certain to be unsuccessful, and will identify a random set of highly expressed (but not differentially regulated) genes. Genes encoding cell wall proteins, ribosomal proteins and other highly expressed genes are well represented in the down-regulated libraries. The high false positive rate in the down-regulated libraries here indicates that the genes observed were not actually down-regulated by MJ.

The gene expression kinetics revealed differential temporal regulation of MJ responsive genes. Up-regulation of many of the MJ induced genes at early time points (6h) was highest, followed by middle time point (18h). MJ-responsive up-regulation of most genes, however, did not persist into the latest time point 5 day (Figure 2). However, a subset of late-induced genes was observed. In agreement to this study, similar expression trend was also observed previously in MJ-induced temporal expression kinetics of taxol biosynthesis genes in P991 cultured cells [27]. This trend was also observed in the paclitaxel-producing *T. cuspidata* P93AF cell line, as semi-quantitative RT-PCR showed that all 12 genes were up-regulated at 6h but transcripts of only 5 of them were detected on day 5. These 12 genes tested here by RT-PCR do not represent any known genes related to taxol biosynthesis, but rather were selected as novel transcripts identified in this study. Furthermore, use of a second paclitaxel-producing cell line (P93AF) demonstrates the reproducibility of the gene expression changes occurring in *Taxus* cultured cells. Paclitaxel and other taxanes accumulate in the cultured cells at time points after 24h [27]. Therefore, persistent translated gene products of early MJ induced genes appear to play a pivotal role in taxane biosynthesis, while late MJ induced genes may be facilitating other functions such as taxane transport and degradation. Genes involved either in intracellular or extracellular paclitaxel transport and degradation in *Taxus* have not yet been characterized [54], but may be present among the ESTs identified here that could not be assigned functions based on molecular similarity to known proteins.

## Conclusions

Several genes were differentially regulated in response to MJ in *Taxus cuspidata* cell lines. Among these genes, paclitaxel biosynthetic pathway genes were specifically induced in response to MJ. A majority of the up-regulated genes followed specific temporal expression kinetics. SSH was not sensitive enough to isolate tightly regulated down regulated genes. Functional characterization of the novel genes identified in this study will further enhance the understanding of paclitaxel metabolism and its regulation.

## Methods

### Cell culture

*Taxu*s *cuspidata* cell line P991 was used for SSH library preparation and macroarray screening and cell line *Taxu*s *cuspidata* P93AF was used for RT-PCR analysis. All cells were obtained as a gift from Dr. D. Gibson at the U.S. Plant Soil and Nutrition laboratory, USDA (Ithaca, NY). Cell cultures were maintained as described in Nims *et al.,* (2006) [27]. MJ (100 μM final concentration) dissolved in 50% EtOH was added to suspension cultures on day 7 after subculture. Control (unelicited) cells were subjected to mock elicitation by adding an equal volume of 50% EtOH. Cells were harvested from elicited and unelicited cultures at 6 hours, 18 hours and 5 day post-elicitation.

### RNA purification

Cells were collected by filtration from the media through Miracloth® (EMD Biosciences, San Diego, CA), flash frozen in liquid nitrogen and stored at -80°C in polypropylene tubes. Total RNA was extracted using guanidinium isothyocyanate and separated through a cesium chloride (CsCl) phase [55]. Poly(A) RNA was extracted using Poly(A) Purist MAG Purification kit (Ambion, Austin, TX). Quality checks of poly (A) RNA preparations were made by PCR using primers specific for 18S rRNA [56]. These primers were: Pt-MGB-18S-f (AGCCTTGCGCTGGCG), Pt-MGB-18S-r (TGCCCTATCAACTTTCGATGGT). Quantification of total RNA and poly(A) RNA was performed with the Ribogreen fluorometric assay (Molecular Probes, Eugene, OR).

### Suppression subtractive hybridization (SSH) libraries

Suppression Subtractive Hybridization (SSH) libraries using control and MJ-elicited samples from *Taxus cuspidata* cell line P991 at 6 hours, 18 hours and 5 day post-elicitation were generated using the Clontech PCR-Select cDNA Subtraction Kit (Takara Bio Inc., Clontech, Mountain View, CA) according to the manufacturer’s instructions. For each SSH library two different double stranded cDNA pools (a ‘tester’ and ‘driver’) were used. Mirror orientation selection (MOS) was then performed to reduce background molecules [53]. Library products were separated on agarose gels. For each library, products below and above 500 base pairs were purified separately using Gel Purification Kit (Qiagen, Valencia, CA) and cloned into TOPO 2-1 vector (Invitrogen, Carlsbad, CA).

### Sequence analysis of the cloned ESTs

Plasmids from independent clones were isolated and single pass sequencing was carried out. All EST sequences were screened through VecScreen software (NCBI: http://www.ncbi.nlm.nih.gov) to remove vector sequence contamination. Once cleaned of vector sequence, all the EST sequences from each up-regulated library and from each down regulated library were used separately for contig assembly with CAP3 [36]. The ESTs were grouped into contigs and singletons and were termed as unigenes (Accession No HE799315-HE799645, Additional file 8: Table S4). The unigene sequences were used to search for homology using the default setting of BLASTX program at NCBI. Each unigene was classified as a protein with known function, a protein with unknown function or a protein with no sequence match in the database. Furthermore, sequences with BLASTx hits were annotated according to gene ontology (GO) terms using Blast2GO software ( http://www.blast2go.org; [57]). The TrEMBL section of UniProtKB ( http://www.uniprot.org) database was used to perform, enzyme class and pathway analysis.

### Expression analysis by macroarray

Macroarrays constructed for this study comprised 256 SSH fragments from both the up-regulated and down-regulated libraries (128 up and 128 down). Bacterial cultures were grown overnight in LB media containing 50 μg/mL kanamycin. Plasmid DNA was purified using Qiaprep-miniprep (Qiagen, Valencia, CA). SSH fragments were amplified by PCR using adapter specific primers. Amplification of fragments via PCR was performed using ExTaq Hot Start Polymerase System (Takara Mirus Bio, Madison, WI). PCR conditions were as follows: 95°C for 2 minutes followed by 25X: 30 seconds, 95°C; 30 seconds, 62°C; 2 minutes, 72°C. This was then followed by 1X: 5 minutes, 72°C. PCR products were precipitated, dissolved in water and denatured in 0.01% sodium dodecyl sulfate (SDS), 0.2 N NaOH at 72°C for 15 minutes. Denatured PCR products were printed in duplicate on Zetaprobe membranes (BioRad, Hercules, CA) using hand held 96-pin replicating tool (V&P Scientific, San Diego, CA). Macroarray blots were submerged in neutralization solution (0.5 M Tris pH 7.4, 1.5 M NaCl), then in 6X sodium citrate/sodium phosphate (SCP) for 1 minute prior to UV cross-linking. Pre-hybridization of the membranes occurred for eight hours in 10% dextran sulfate (average molecular weight of 500,000kD; G.E. Health, Piscataway, NJ), 5.5X SCP, 0.9% *n*-lauroyl sulfate-sodium salt and 0.5 mg/ml heparin. Targets were labelled with ^32^P dCTP (G.E. Healthcare Bio-Sciences, Piscataway, NJ) using Superscript™ II Reverse Transcriptase (RT) (Invitrogen, Carlsbad, CA) from 300 ng polyA RNA. Hybridization was performed at 65°C for 18H, followed by washing twice with 2X SCP, 0.1% SDS for 15 minutes then twice using 0.2X SCP and 0.2% SDS for 15 minutes at 65°C. Blots were exposed to phosphorimager screen for 24-48H then read by FLA-5000 phosphorimager (Fujifilm Medical Systems Inc., Stamford, CT). Probes were stripped off of blots up to three times using 0.1% SDS at 100°C. Blots were checked for residual hybridization by exposing to film for 24H before re-hybridizing with a different target cDNA.

Signal intensities of each spot were captured using VisualGrid (GPC Biotech Inc, Waltham, MA). Following local background subtraction, the intensity values for each spot were globally normalized using LOWESS (locally weighted polynomial regression [58]) within and across all four technical replicate experiments. The relative fold change expression signal obtained from each clone found significant in one tailed student t-test in response to MJ over mock elicitation were considered for further analysis (≥ 2 fold for up and down). Additionally, relative fold change of three or more independent ESTs corresponding to a particular contig were analysed using Q-test with 95% confidence for finding outlying data points.

### Gene expression analysis by RT-PCR

Total RNA was isolated from *Taxus cuspidata* P93AF cell line at 6h, 18h and 5 day after MJ elicitation as well as from unelicited cells (18 h) using RNeasy Plant Mini kit (Qiagen). First strand cDNA was synthesized using Affinityscript™ cDNA synthesis kit (Stratagene) from 3μg total RNA following the manufacture’s protocol. Primers ( Additional file 9: Table S5) for each subtracted cDNA were designed using Primer3 ( http://fokker.wi.mit.edu/primer3/input.htm). A touchdown PCR was performed for all the transcripts with annealing temperature varying from 64°C to 52°C. The number of cycles used to amplify each fragment was variable in order to detect the corresponding amplicon during linear amplification.

## Competing interests

The authors declare that they have no competing interests.

## Authors' contributions

SKL analysed the data and drafted the manuscript; NB created the SSH library and performed macroarray analysis; RT-PCR analysis was carried out by BP; KV prepared the cell cultures and JN, SCR and ELW designed and mentored the study and gave final approval to the manuscript. All authors read and approved the final manuscript.

## Supplementary Material

Additional file 1 **Table S1.** The duplicate sequences obtained from the three up-regulated libraries corresponding to each contig. Click here for file

Additional file 2 **Table S2.** Identity and description of unigenes derived from up- and down- regulated *T. cuspidata SSH libraries.* Click here for file

Additional file 3 **Figure S1.** Gene Ontology mapping. GO mapping for *Taxus cuspidata* up-regulated unigenes by (**a**) biological process, (**b**) molecular function, and (**c**) cellular components. Click here for file

Additional file 4 **Table S3.** Macroarray data from both the up- and down-regulated libraries hybridised with various RNA targets. Click here for file

Additional file 5 **Figure S2.** Pattern of expression of clones from down-regulated libraries. Macroarrays spotted with randomly selected clones (probes) from all six SSH libraries were hybridized with labelled RNA targets prepared from mock elicited and elicited cells at three time points: 6h, 18h, and 5 day. Expression profile plots for the set of probes corresponding to each down-regulated library are shown: 6h library probes. Expression is given as the fold-change in gene expression in the elicited culture over the un-elicited culture. Only results from probes showing a statistically significant change in expression (P < 0.05) are shown. Click here for file

Additional file 6 **Figure S3.** Pattern of expression of clones from down-regulated libraries. Macroarrays spotted with randomly selected clones (probes) from all six SSH libraries were hybridized with labelled RNA targets prepared from mock elicited and elicited cells at three time points: 6h, 18h, and 5 day. Expression profile plots for the set of probes corresponding to each down-regulated library are shown: 18h library probes **C**. Expression is given as the fold-change in gene expression in the elicited culture over the un-elicited culture. Only results from probes showing a statistically significant change in expression (P < 0.05) are shown. Click here for file

Additional file 7 **Figure S4.** Pattern of expression of clones from down-regulated libraries. Macroarrays spotted with randomly selected clones (probes) from all six SSH libraries were hybridized with labelled RNA targets prepared from mock elicited and elicited cells at three time points: 6h, 18h, and 5 day. Expression profile plots for the set of probes corresponding to each down-regulated library are shown: 5 day library probes. Expression is given as the fold-change in gene expression in the elicited culture over the un-elicited culture. Only results from probes showing a statistically significant change in expression (P < 0.05) are shown. Click here for file

Additional file 8 **Table S4.** List of sequences obtained from the SSH libraries. Click here for file

Additional file 9 **Table S5.** List of primer sets used for RT-PCR analysis. Click here for file

## References

[B1] Farnsworth NR Akerele O Bingel AS Soejarto DD Guo Z Medicinal plants in therapy Bull World Health Organ 1985 63 6 965 981 3879679 PMC2536466

[B2] Exposito O Bonfill M Moyano E Onrubia M Mirjalili MH Cusido RM Palazon J Biotechnological production of taxol and related taxoids: current state and prospects Anticancer Agents Med Chem 2009 9 1 109 121 19149486 10.2174/187152009787047761

[B3] Vongpaseuth K Roberts SC Advancements in the understanding of Paclitaxel metabolism in tissue culture Curr Pharm Biotechnol 2007 8 4 219 236 17691991 10.2174/138920107781387393

[B4] Frense D Taxanes: perspectives for biotechnological production Appl Microbiol Biotechnol 2007 73 6 1233 1240 10.1007/s00253-006-0711-0 17124581

[B5] Tabata H Production of paclitaxel and the related taxanes by cell suspension cultures of Taxus species Curr Drug Targets 2006 7 4 453 461 10.2174/138945006776359368 16611032

[B6] Kolewe ME Henson MA Roberts SC Characterization of aggregate size in Taxus suspension cell culture Plant Cell Rep 2010 29 5 485 494 10.1007/s00299-010-0837-5 20217417 PMC4339049

[B7] Vongpaseuth K Nims E St Amand M Walker EL Roberts SC Development of a particle bombardment-mediated transient transformation system for Taxus spp. cells in culture Biotechnol Prog 2007 23 5 1180 1185 17725365 10.1021/bp0700307

[B8] Naill MC Roberts SC Culture of isolated single cells from Taxus suspensions for the propagation of superior cell populations Biotechnol Lett 2005 27 21 1725 1730 10.1007/s10529-005-2738-1 16247682

[B9] Roberts SC Naill M Gibson DM Shuler ML A simple method for enhancing paclitaxel release from Taxus canadensis cell suspension cultures utilizing cell wall digesting enzymes Plant Cell Rep 2003 21 12 1217 1220 10.1007/s00299-003-0575-z 12811504

[B10] Pestchanker LJ Roberts SC Shuler ML Kinetics of taxol production and nutrient use in suspension cultures of Taxus cuspidata in shake flasks and a Wilson-type bioreactor Enzyme Microb Technol 1996 19 4 256 260 10.1016/0141-0229(95)00243-X 8987485

[B11] Fett-Neto AG Melanson SJ Nicholson SA Pennington JJ Dicosmo F Improved taxol yield by aromatic carboxylic acid and amino acid feeding to cell cultures of taxus cuspidata Biotechnol Bioeng 1994 44 8 967 971 10.1002/bit.260440813 18618915

[B12] Zhang MLX Zhang J Zhang S Dong M Huo C Shi Q Gu Y Cong B Taxanes from the leaves of Taxus cuspidata Chem Nat Comp 2010 46 1 53 58 10.1007/s10600-010-9523-7

[B13] Wani MC Taylor HL Wall ME Coggon P McPhail AT Plant antitumor agents. VI. The isolation and structure of taxol, a novel antileukemic and antitumor agent from Taxus brevifolia J Am Chem Soc 1971 93 9 2325 2327 10.1021/ja00738a045 5553076

[B14] Hezari M Ketchum RE Gibson DM Croteau R Taxol production and taxadiene synthase activity in Taxus canadensis cell suspension cultures Arch Biochem Biophys 1997 337 2 185 190 10.1006/abbi.1996.9772 9016812

[B15] Walker K Croteau R Taxol biosynthetic genes Phytochemistry 2001 58 1 1 7 10.1016/S0031-9422(01)00160-1 11524108

[B16] Hefner J Rubenstein SM Ketchum RE Gibson DM Williams RM Croteau R Cytochrome P450-catalyzed hydroxylation of taxa-4(5),11(12)-diene to taxa-4(20),11(12)-dien-5alpha-ol: the first oxygenation step in taxol biosynthesis Chem Biol 1996 3 6 479 489 10.1016/S1074-5521(96)90096-4 8807878

[B17] Hefner J Ketchum RE Croteau R Cloning and functional expression of a cDNA encoding geranylgeranyl diphosphate synthase from Taxus canadensis and assessment of the role of this prenyltransferase in cells induced for taxol production Arch Biochem Biophys 1998 360 1 62 74 10.1006/abbi.1998.0926 9826430

[B18] Walker K Croteau R Molecular cloning of a 10-deacetylbaccatin III-10-O-acetyl transferase cDNA from Taxus and functional expression in Escherichia coli Proc Natl Acad Sci U S A 2000 97 2 583 587 10.1073/pnas.97.2.583 10639122 PMC15373

[B19] Wildung MR Croteau R A cDNA clone for taxadiene synthase, the diterpene cyclase that catalyzes the committed step of taxol biosynthesis J Biol Chem 1996 271 16 9201 9204 10.1074/jbc.271.16.9201 8621577

[B20] Schoendorf A Rithner CD Williams RM Croteau RB Molecular cloning of a cytochrome P450 taxane 10 beta-hydroxylase cDNA from Taxus and functional expression in yeast Proc Natl Acad Sci U S A 2001 98 4 1501 1506 10.1073/pnas.98.4.1501 11171980 PMC29286

[B21] Walker K Long R Croteau R The final acylation step in taxol biosynthesis: cloning of the taxoid C13-side-chain N-benzoyltransferase from Taxus Proc Natl Acad Sci U S A 2002 99 14 9166 9171 10.1073/pnas.082115799 12089320 PMC123112

[B22] Walker K Fujisaki S Long R Croteau R Molecular cloning and heterologous expression of the C-13 phenylpropanoid side chain-CoA acyltransferase that functions in Taxol biosynthesis Proc Natl Acad Sci U S A 2002 99 20 12715 12720 10.1073/pnas.192463699 12232048 PMC130526

[B23] Jennewein S Rithner CD Williams RM Croteau RB Taxol biosynthesis: taxane 13 alpha-hydroxylase is a cytochrome P450-dependent monooxygenase Proc Natl Acad Sci U S A 2001 98 24 13595 13600 10.1073/pnas.251539398 11707604 PMC61086

[B24] Walker K Croteau R Taxol biosynthesis: molecular cloning of a benzoyl-CoA:taxane 2alpha-O-benzoyltransferase cDNA from taxus and functional expression in Escherichia coli Proc Natl Acad Sci U S A 2000 97 25 13591 13596 10.1073/pnas.250491997 11095755 PMC17620

[B25] Walker K Schoendorf A Croteau R Molecular cloning of a taxa-4(20),11(12)-dien-5alpha-ol-O-acetyl transferase cDNA from Taxus and functional expression in Escherichia coli Arch Biochem Biophys 2000 374 2 371 380 10.1006/abbi.1999.1609 10666320

[B26] Gundlach H Muller MJ Kutchan TM Zenk MH Jasmonic acid is a signal transducer in elicitor-induced plant cell cultures Proc Natl Acad Sci U S A 1992 89 6 2389 2393 10.1073/pnas.89.6.2389 11607285 PMC48663

[B27] Nims E Dubois CP Roberts SC Walker EL Expression profiling of genes involved in paclitaxel biosynthesis for targeted metabolic engineering Metab Eng 2006 8 5 385 394 10.1016/j.ymben.2006.04.001 16793302

[B28] Kai G Zhao L Zhang L Li Z Guo B Zhao D Sun X Miao Z Tang K Characterization and expression profile analysis of a new cDNA encoding taxadiene synthase from Taxus media J Biochem Mol Biol 2005 38 6 668 675 10.5483/BMBRep.2005.38.6.668 16336781

[B29] Naill MC Roberts SC Flow cytometric analysis of protein content in Taxus protoplasts and single cells as compared to aggregated suspension cultures Plant Cell Rep 2005 23 8 528 533 10.1007/s00299-004-0875-y 15449019

[B30] Yukimune Y Tabata H Higashi Y Hara Y Methyl jasmonate-induced overproduction of paclitaxel and baccatin III in Taxus cell suspension cultures Nat Biotechnol 1996 14 9 1129 1132 10.1038/nbt0996-1129 9631065

[B31] Yukimune Y Hara Y Nomura E Seto H Yoshida S The configuration of methyl jasmonate affects paclitaxel and baccatin III production in Taxus cells Phytochemistry 2000 54 1 13 17 10.1016/S0031-9422(00)00006-6 10846740

[B32] Wu Q Sun C Luo H Li Y Niu Y Sun Y Lu A Chen S Transcriptome analysis of Taxus cuspidata needles based on 454 pyrosequencing Planta Med 2011 77 4 394 400 10.1055/s-0030-1250331 20862637

[B33] da Hao C Ge G Xiao P Zhang Y Yang L The First Insight into the Tissue Specific Taxus Transcriptome via Illumina Second Generation Sequencing PLoS One 2011 6 6 e21220 10.1371/journal.pone.0021220 21731678 PMC3120849

[B34] Lee EK Jin YW Park JH Yoo YM Hong SM Amir R Yan Z Kwon E Elfick A Tomlinson S Cultured cambial meristematic cells as a source of plant natural products Nat Biotechnol 2010 28 11 1213 1217 10.1038/nbt.1693 20972422

[B35] Naill MC Roberts SC Flow cytometric identification of Paclitaxel-accumulating subpopulations Biotechnol Prog 2005 21 3 978 983 15932283 10.1021/bp049544l

[B36] Huang X Madan A CAP3: A DNA sequence assembly program Genome Res 1999 9 9 868 877 10.1101/gr.9.9.868 10508846 PMC310812

[B37] Ketchum REB Gibson DM Croteau RB Shuler ML The kinetics of taxoid accumulation in cell suspension cultures of *Taxus* following elicitation with methyl jasmonate Biotechnol Bioeng 1999 62 1 97 105 10.1002/(SICI)1097-0290(19990105)62:1<97::AID-BIT11>3.0.CO;2-C 10099517

[B38] Mirjalili N Linden JC Methyl jasmonate induced production of Taxol in suspension cultures of *Taxus cuspidata*: Ethylene interaction and induction models Biotechnol Prog 1996 12 110 118 10.1021/bp9500831 8845101

[B39] Yukimune Y Tabata H Higashi Y Hara Y Methyl Jasmonate-induced overproduction of paclitaxel and baccatin III in *Taxus* cell suspension cultures Nat Biotechnol 1996 14 1129 1132 10.1038/nbt0996-1129 9631065

[B40] Kim BJ Gibson DM Shuler ML Relationship of viability and apoptosis to taxol production in Taxus sp. suspension cultures elicited with methyl jasmonate Biotechnol Prog 2005 21 3 700 707 15932245 10.1021/bp050016z

[B41] Weber H Fatty acid-derived signals in plants Trends Plant Sci 2002 7 217 224 10.1016/S1360-1385(02)02250-1 11992827

[B42] Hermsmeier D Schittko U Baldwin IT Molecular interactions between the specialist herbivore *Manduca sexta* (Lepidoptera, Sphingidae) and its natural host *Nicotiana attenuata*.I. Large-scale changes in accumulation of growth- and defenese-related plant mRNAs Plant Physiol 2001 125 683 700 10.1104/pp.125.2.683 11161026 PMC64870

[B43] Croteau R Ketchum REB Long RM Kaspera R Wildung MR Taxol biosynthesis and molecular genetics Phytochem Rev 2006 5 75 97 10.1007/s11101-005-3748-2 20622989 PMC2901146

[B44] Ketchum REB Croteau R Saito KD, Dixon RA, Willmitzer L The Taxus metabolome and the elucidation of the Taxol biosynthetic pathway in cell suspension cultures Biotechnology in Agriculture and Forestry 2006 Springer-Verlag, Berlin 291 309

[B45] Chau M Croteau R Molecular cloning and characterization of a cytochrome P450 taxoid 2alpha-hydroxylase involved in Taxol biosynthesis Arch Biochem Biophys 2004 427 1 48 57 10.1016/j.abb.2004.04.016 15178487

[B46] Walker K Ketchum RE Hezari M Gatfield D Goleniowski M Barthol A Croteau R Partial purification and characterization of acetyl coenzyme A: taxa-4(20),11(12)-dien-5alpha-ol O-acetyl transferase that catalyzes the first acylation step of taxol biosynthesis Arch Biochem Biophys 1999 364 2 273 279 10.1006/abbi.1999.1125 10190984

[B47] Spanu P Reinhart D Boller T Analysis and cloning of the ethylene-forming enzyme from tomato by functional expression of its mRNA in Xenopus laevis oocytes EMBO J 1991 10 2007 2013 2065651 10.1002/j.1460-2075.1991.tb07730.x PMC452880

[B48] Zhang CH Wu JY Ethylene inhibitors enhance elicitor-induced paclitaxel production in suspension cultures of Taxus spp. cells Enzyme Microb Technol 2003 32 71 77 10.1016/S0141-0229(02)00266-1

[B49] van Loon LC Rep M Pieterse CM Significance of inducible defense-related proteins in infected plants Annu Rev Phytopathol 2006 44 135 162 10.1146/annurev.phyto.44.070505.143425 16602946

[B50] Lamb CJ Dixon RA Molecular mechanisms underlying induction of plant defence gene transcription Biochem Soc Symp 1994 60 241 248 7639783

[B51] Ji SJ Lu YC Feng JX Wei G Li J Shi YH Fu Q Liu D Luo JC Zhu YX Isolation and analyses of genes preferentially expressed during early cotton fiber development by subtractive PCR and cDNA array Nucleic Acids Res 2003 31 10 2534 2543 10.1093/nar/gkg358 12736302 PMC156040

[B52] Zheng J Zhao J Tao Y Wang J Liu Y Fu J Jin Y Gao P Zhang J Bai Y Isolation and analysis of water stress induced genes in maize seedlings by subtractive PCR and cDNA macroarray Plant Mol Biol 2004 55 6 807 823 15604718 10.1007/s11103-004-1969-1

[B53] Rebrikov DV Britanova OV Gurskaya NG Lukyanov KA Tarabykin VS Lukyanov SA Mirror orientation selection (MOS): a method for eliminating false positive clones from libraries generated by suppression subtractive hybridization Nucleic Acids Res 2000 28 20 E90 10.1093/nar/28.20.e90 11024192 PMC110806

[B54] Roberts SC Production and engineering of terpenoids in plant cell culture Nat Chem Biol 2007 3 7 387 395 10.1038/nchembio.2007.8 17576426

[B55] Chirgwin JM Przybyla AE MacDonald RJ Rutter WJ Isolation of biologically active ribonucleic acid from sources enriched in ribonuclease Biochemistry 1979 18 24 5294 5299 10.1021/bi00591a005 518835

[B56] Dilger M Felsenstein FG Schwarz G Identification and quantitative expression analysis of genes that are differentially expressed during conidial germination in Pyrenophora teres Mol Genet Genomics 2003 270 2 147 155 10.1007/s00438-003-0910-7 12938040

[B57] Conesa A Gotz S Garcia-Gomez JM Terol J Talon M Robles M Blast2GO: a universal tool for annotation, visualization and analysis in functional genomics research Bioinformatics 2005 21 18 3674 3676 10.1093/bioinformatics/bti610 16081474

[B58] Cleveland W Robust locally weighted regression and smoothing scatter plots J Am Stat Assoc 1979 74 368 829 836 10.1080/01621459.1979.10481038

