# Supplementary material for: Identification and expression analysis of methyl jasmonate responsive ESTs in paclitaxel producing Taxus cuspidata suspension culture cells
Source: BMC Genomics. 2012 Apr 24;13:148. doi: 10.1186/1471-2164-13-148 (PMC3489508; doi:10.1186/1471-2164-13-148)
Supplement: Additional file 1 — Table S1. The duplicate sequences obtained from the three up-regulated libraries corresponding to each contig. [file 1471-2164-13-148-S1.doc]

**Supplemental Table 1:** The duplicate sequences obtained from the three up-regulated libraries corresponding to each contig

| Contig no | No. of clones from | | |
| --- | --- | --- | --- |
|  | 6h | 18h | 5D |
| 1 | 2 | 0 | 0 |
| 2 | 15 | 1 | 1 |
| 3 | 11 | 4 | 0 |
| 4 | 3 | 0 | 0 |
| 5 | 2 | 0 | 0 |
| 6 | 0 | 4 | 1 |
| 7 | 0 | 3 | 2 |
| 8 | 0 | 4 | 0 |
| 9 | 0 | 2 | 0 |
| 10 | 0 | 3 | 0 |
| 11 | 0 | 3 | 7 |
| 12 | 0 | 2 | 0 |
| 13 | 0 | 4 | 0 |
| 14 | 0 | 2 | 2 |
| 15 | 0 | 5 | 9 |
| 16 | 0 | 2 | 0 |
| 17 | 2 | 1 | 0 |
| 18 | 1 | 1 | 0 |
| 19 | 0 | 2 | 0 |
| 20 | 0 | 0 | 5 |
| 21 | 0 | 0 | 3 |
| 22 | 0 | 1 | 7 |
| 23 | 0 | 0 | 4 |
| 24 | 0 | 0 | 9 |
| 25 | 0 | 0 | 6 |
| 26 | 0 | 0 | 2 |
| 27 | 0 | 0 | 2 |
| 28 | 0 | 0 | 8 |
| 29 | 0 | 0 | 2 |
| 30 | 0 | 0 | 2 |
| 31 | 0 | 0 | 2 |
| 32 | 0 | 0 | 3 |
