# Supplementary material for: Identification and expression analysis of methyl jasmonate responsive ESTs in paclitaxel producing Taxus cuspidata suspension culture cells
Source: BMC Genomics. 2012 Apr 24;13:148. doi: 10.1186/1471-2164-13-148 (PMC3489508; doi:10.1186/1471-2164-13-148)
Supplement: Additional file 3 — Figure S1. Gene Ontology mapping. GO mapping for Taxus cuspidata up-regulated unigenes by (a) biological process, (b) molecular function, and (c) cellular components. [file 1471-2164-13-148-S3.ppt]

## Slide 1
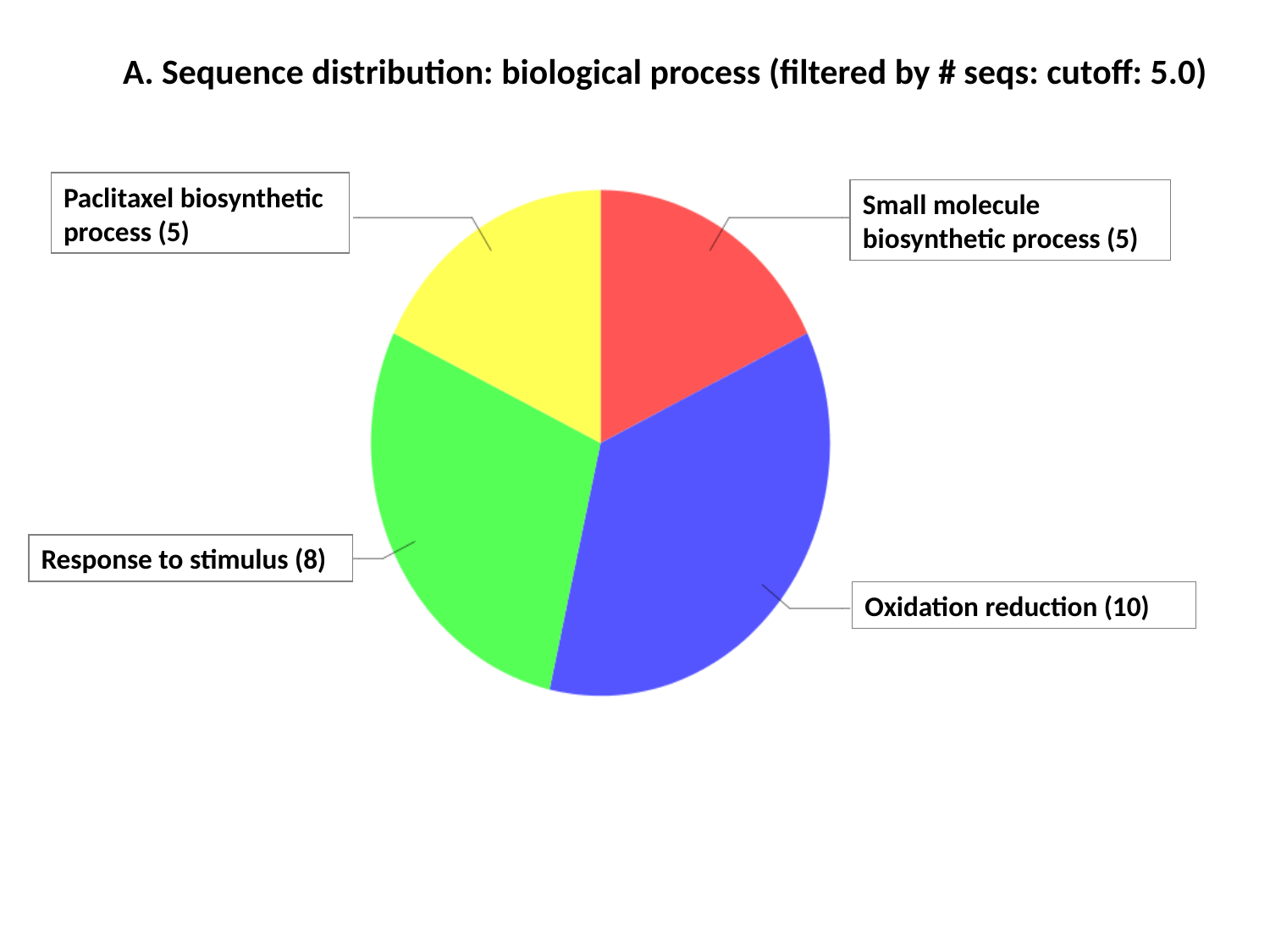

A. Sequence distribution: biological process (filtered by # seqs: cutoff: 5.0)
Paclitaxel biosynthetic process (5)
Small molecule biosynthetic process (5)
Response to stimulus (8)
Oxidation reduction (10)

## Slide 2
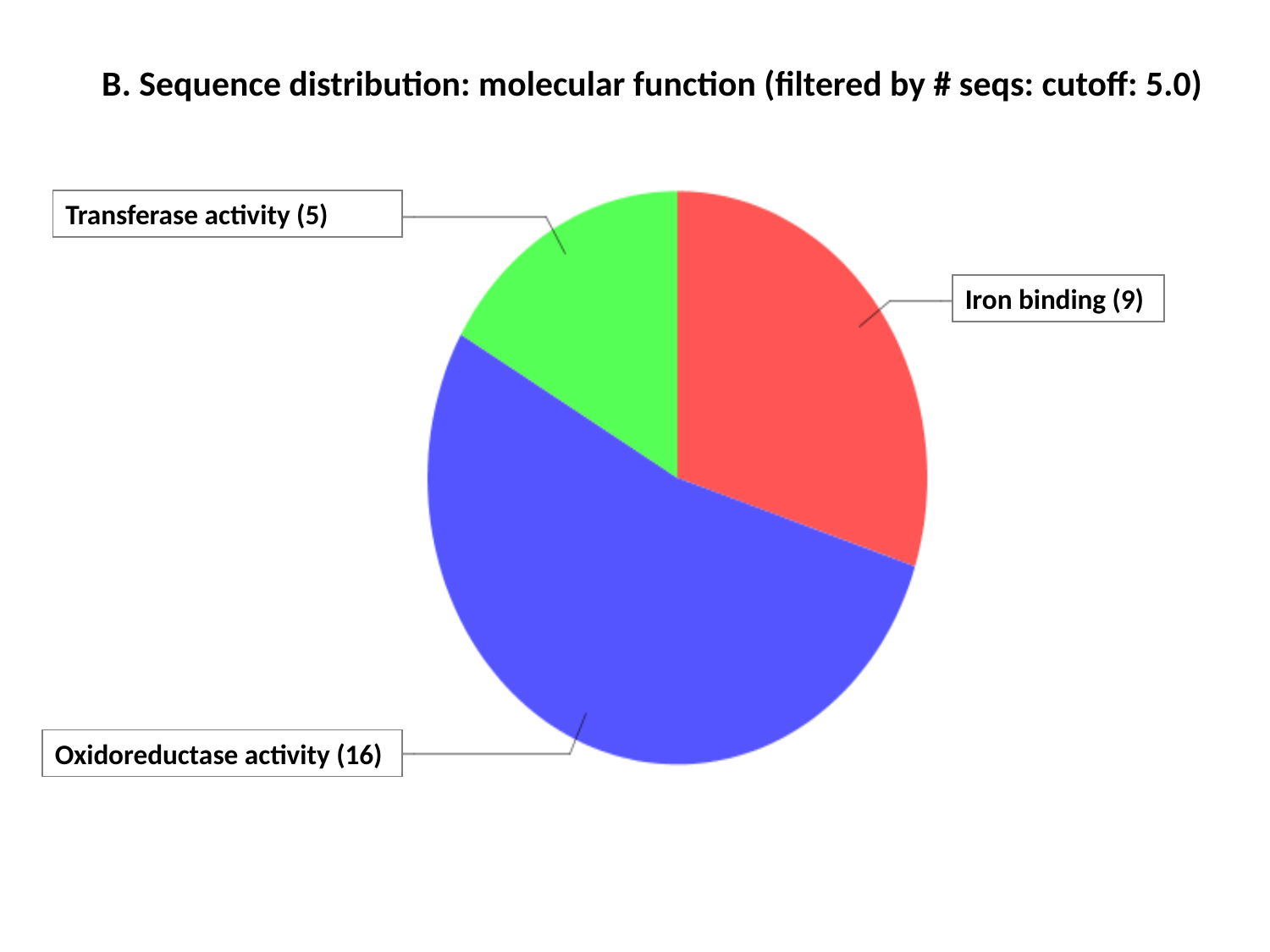

B. Sequence distribution: molecular function (filtered by # seqs: cutoff: 5.0)
Transferase activity (5)
Iron binding (9)
Oxidoreductase activity (16)

## Slide 3
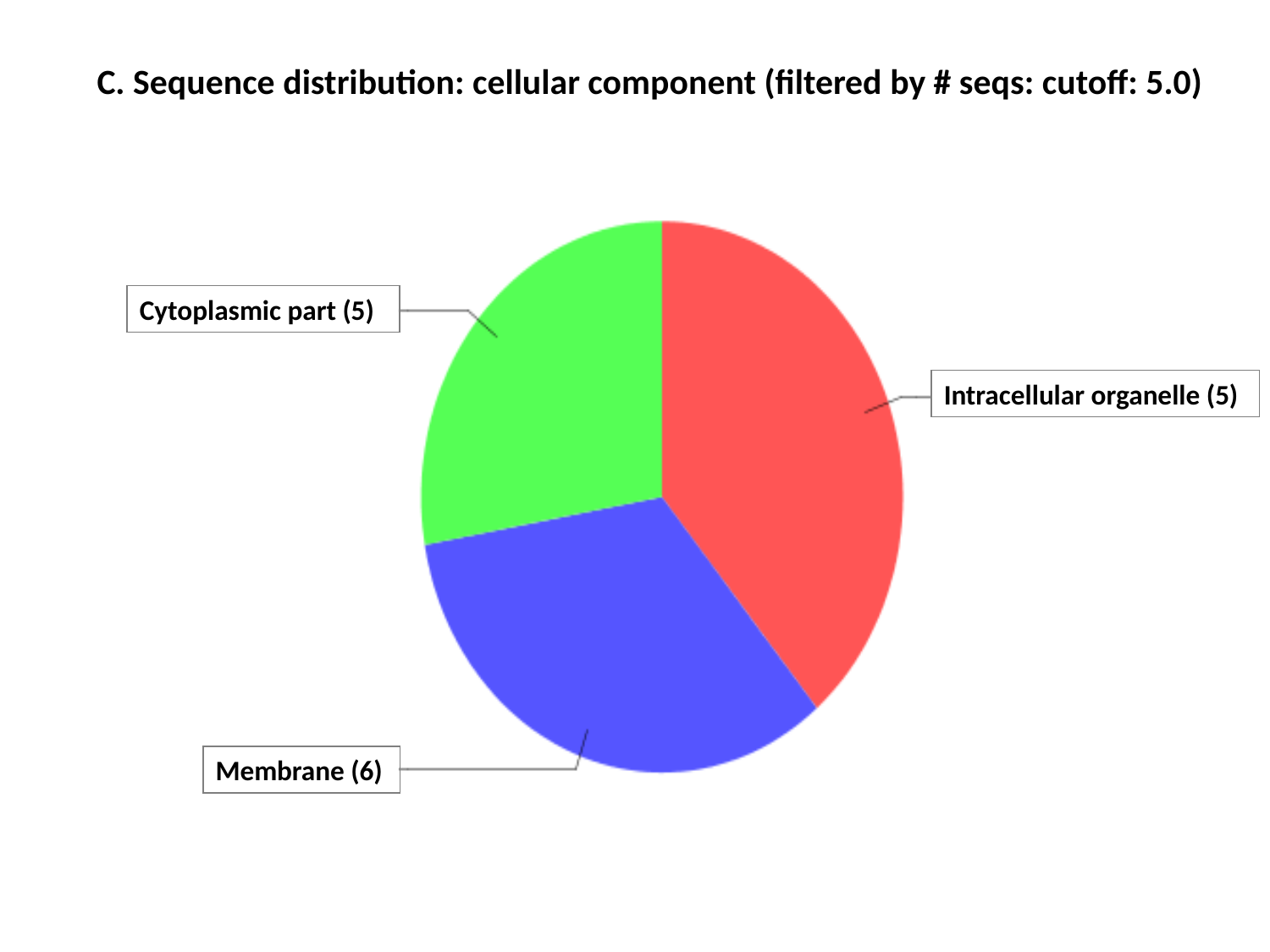

C. Sequence distribution: cellular component (filtered by # seqs: cutoff: 5.0)
Cytoplasmic part (5)
Intracellular organelle (5)
Membrane (6)
