# Supplementary figures and images for: Identification and expression analysis of methyl jasmonate responsive ESTs in paclitaxel producing Taxus cuspidata suspension culture cells
Source: BMC Genomics. 2012 Apr 24;13:148. doi: 10.1186/1471-2164-13-148 (PMC3489508; doi:10.1186/1471-2164-13-148)

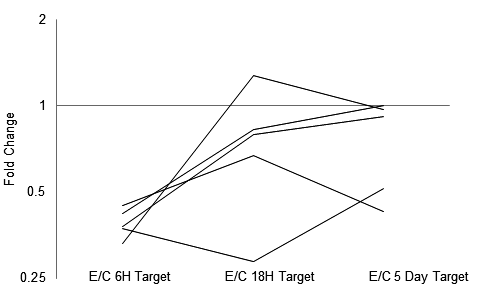

Supplement: Additional file 5 — Figure S2. Pattern of expression of clones from down-regulated libraries. Macroarrays spotted with randomly selected clones (probes) from all six SSH libraries were hybridized with labelled RNA targets prepared from mock elicited and elicited cells at three time points: 6h, 18h, and 5 day. Expression profile plots for the set of probes corresponding to each down-regulated library are shown: 6h library probes. Expression is given as the fold-change in gene expression in the elicited culture over the un-elicited culture. Only results from probes showing a statistically significant change in expression (P < 0.05) are shown. [file 1471-2164-13-148-S5.png]

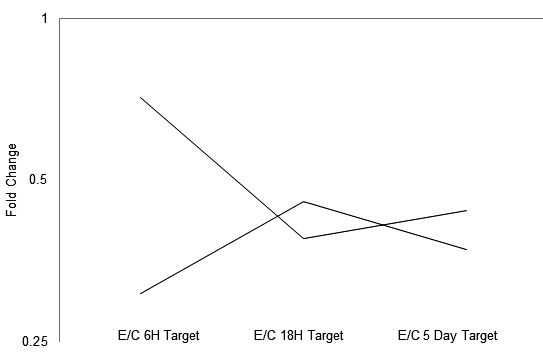

Supplement: Additional file 6 — Figure S3. Pattern of expression of clones from down-regulated libraries. Macroarrays spotted with randomly selected clones (probes) from all six SSH libraries were hybridized with labelled RNA targets prepared from mock elicited and elicited cells at three time points: 6h, 18h, and 5 day. Expression profile plots for the set of probes corresponding to each down-regulated library are shown: 18h library probes C. Expression is given as the fold-change in gene expression in the elicited culture over the un-elicited culture. Only results from probes showing a statistically significant change in expression (P < 0.05) are shown. [file 1471-2164-13-148-S6.png]

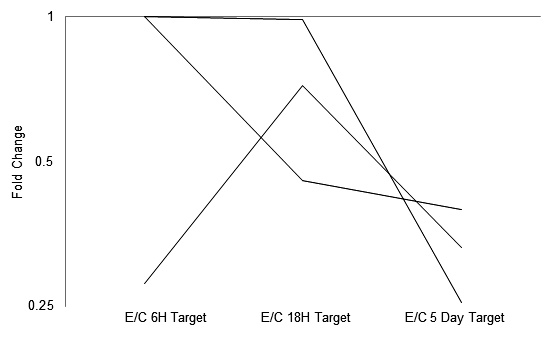

Supplement: Additional file 7 — Figure S4. Pattern of expression of clones from down-regulated libraries. Macroarrays spotted with randomly selected clones (probes) from all six SSH libraries were hybridized with labelled RNA targets prepared from mock elicited and elicited cells at three time points: 6h, 18h, and 5 day. Expression profile plots for the set of probes corresponding to each down-regulated library are shown: 5 day library probes. Expression is given as the fold-change in gene expression in the elicited culture over the un-elicited culture. Only results from probes showing a statistically significant change in expression (P < 0.05) are shown. [file 1471-2164-13-148-S7.png]
