# Supplementary material for: Identification and expression analysis of methyl jasmonate responsive ESTs in paclitaxel producing Taxus cuspidata suspension culture cells
Source: BMC Genomics. 2012 Apr 24;13:148. doi: 10.1186/1471-2164-13-148 (PMC3489508; doi:10.1186/1471-2164-13-148)
Supplement: Additional file 9 — Table S5. List of primer sets used for RT-PCR analysis. [file 1471-2164-13-148-S9.doc]

**Supplemental Table 5: List of primer sets used for RT-PCR analysis**

| Cytochrome P450 (contig 3) F | ATGTGTCCGGGCATGAGT |
| --- | --- |
| Cytochrome P450 (contig 3) R | GGGAAGGCGAGGTTTGAT |
| Quinone oxidoreductase (SU25) F | GGTGGTCTCCACCACGAG |
| Quinone oxidoreductase (SU25) R | TGGGTGCTCCTTCTTTCG |
| Unknown (contig 6) F | GGTACTCCAGCGCAAAACA |
| Unknown (contig 6) R | CGCCGGAGCACATATTTC |
| Hypothetical protein (contig 16) F | CCTGCCTCGCCTATCTCA |
| Hypothetical protein (contig 16) R | TGGCCCAATCTCCCAATA |
| Dirigent-like protein ( SU3 ) F | CAATGGCCAAAATGCTCA |
| Dirigent-like protein ( SU3 ) R | ACTGGAGGAGAGCGCAGA |
| Lipoxygenase (contig 18) F | CCAGGCGCTTTATTCCAG |
| Lipoxygenase (contig 18) R | GTGGATCCCCTGCAACTG |
| Leucoanthocyanidin reductase (contig 26) F | GTGGACGACCCACGAACT |
| Leucoanthocyanidin reductase (contig 26) R | GGCCACAATGCTTGAAGG |
| ACC oxidase (contig 9) F | AGGCAAACGATCGAGGAA |
| ACC oxidase (contig 9) R | GGGCAGGGAGGATAATGG |
| Pyridine nucleotide-disulfide oxidoreductase family protein ( SU30 ) F | AAAGCAGCGGGAATAGCA |
| Pyridine nucleotide-disulfide oxidoreductase family protein ( SU30 ) R | GCCTTCCACCCCTCAGAT |
| Cytochromep450 (contig 32) F | GGGGATTACCATGGTGGAA |
| Cytochromep450 (contig 32) R | GGCGAGGGGTAGCCTTG |
| Flavonol synthase ( SU5) F | GCTTGCTCTGGGAGTGGA |
| Flavonol synthase ( SU5) R | AGCTGGTCTCCGATGTGG |
| Short-chain dehydrogenase/reductase family protein ( SU15 ) F | CGCGAATACACCAGAGCA |
| Short-chain dehydrogenase/reductase family protein (SU15 ) R | GGCGGCATAGCTCCGTAT |
